# Comparison of Dilution on Eastern Box Turtle (*Terrapene carolina carolina*) and Marine Toad *(Rhinella marinus*) Blood Parameters as Measured on a Portable Chemistry Analyzer

**DOI:** 10.1155/2020/8843058

**Published:** 2020-08-27

**Authors:** John A. Griffioen, Devorah M. Stowe, Macy Trosclair, Larry J. Minter, Chelsey Vanetten, Tara M. Harrison

**Affiliations:** ^1^Department of Clinical Services, College of Veterinary Medicine, North Carolina State University, Raleigh, NC 27607, USA; ^2^Veterinary Department, Indianapolis Zoo, Indianapolis, IN 46222, USA; ^3^Department of Population Health and Pathobiology, College of Veterinary Medicine, North Carolina State University, Raleigh, NC 27607, USA; ^4^Natick Animal Clinic, Natick, MA 01760, USA; ^5^Hanes Veterinary Medical Center, North Carolina Zoo, 4401 Zoo Parkway, Asheboro, NC 27205, USA

## Abstract

Biochemical testing is an important clinical tool in evaluating the physiology of reptiles and amphibians. Suitable point of care analyzers can allow for rapid delivery of results, but small patient size can inhibit sufficient sample collection. This study evaluated the utility of sample dilution with sterile distilled water as a means of biochemical evaluation when sample volume is limited. Blood was collected from 12 eastern box turtles (*Terrapene carolina carolina*) and 12 marine toads *(Rhinella marinus*) and analyzed via i-STAT CHEM8+ cartridges. Two undiluted samples and two samples diluted 1 : 1 with sterile water were evaluated immediately following collection for each animal in the study. Analytes reported in the diluted samples were limited to glucose, ionized calcium, and total carbon dioxide. The expected dilution ratio value of diluted to undiluted samples was 0.5, of which glucose in both turtles and toads was nearest. Dilution ratio values for ionized calcium, however, were higher than expected in both turtles and toads. Sample dilution is not recommended for most analytes included on the CHEM8+ cartridge due to values occurring outside the limits of detection for the analyzer. Glucose and ionized calcium values obtained on diluted samples should be interpreted with caution but may provide clinical utility in reptile and amphibian patients where sample volume is limited.

## 1. Introduction

Biochemical testing is utilized by veterinarians across disciplines to contribute to a complete clinical examination in the health management of animals. Point of care testing using portable analyzers is common in veterinary medicine to measure complete blood counts and plasma or serum chemistry analytes, as well as circulating hormones, drug residues, and detection of specific infectious diseases in some species [1–4]. In reptile and amphibian species, analyzers such as the i-STAT Portable Clinical Analyzer (Abbott Point of Care Inc., Princeton, NJ 08540, USA) have been used for the measurement of blood gases and biochemical parameters [2, 5–7]. The i-STAT analyzer has also been employed in field research studies in reptiles where benchtop analysis was unavailable or unfeasible [8–11].

An analyte of particular importance in the clinical assessment of many reptiles and amphibians is plasma or serum ionized calcium. Circulating ionized calcium remains the most reliable indicator of physiologically active calcium, irrespective of the animal's age or sex, and therefore is an essential part of therapeutic planning in diseases altering calcium homeostasis [12]. Metabolic bone diseases (MBDs), a common group of diseases in many captive reptiles and amphibians, typically present as nutritional secondary hyperparathyroidism (NSHPT) and renal secondary hyperparathyroidism (RSHPT) [13]. Measurement of ionized calcium values is especially useful in the diagnosis and management of these MBDs [14].

In some animals, however, even the relatively small blood volume required for point of care analyzers such as the i-STAT (0.095 mL) is higher than what is regarded as a safe collection volume. In amphibians, blood volume is reported to be approximately 10% body weight, with a safe blood collection volume of 10% of total blood volume, or 1% body weight in most animals [15, 16]. Given the reported volume recommendations and the necessary volume for analysis, a sufficient amount of blood could not be collected from amphibians under 10 grams of body weight. In reptiles, blood volume is reported to be lower at approximately 5–8% body weight, with a safe blood collection volume of 10% of total blood volume, or 0.5–0.8% body weight in most animals [17–19]. Therefore, in reptiles weighing less than 12.5–20 grams, safety concerns would preclude obtaining a diagnostic sample.

A previous study examined the accuracy of diluting avian blood samples with sterile water at a variety of dilutions and found that the values were overall unreliable, and therefore, they did not recommend diluting samples to increase sample volume [20]. However, this study utilized a benchtop analyzer, avian blood, and did not evaluate ionized calcium values. The purpose of this study was to evaluate the feasibility and accuracy of measuring specific blood parameters, including ionized calcium, in small reptile and amphibian patients by comparing undiluted and diluted samples on a point of care analyzer. It was hypothesized that the majority of blood parameters would be diluted to levels below the limit of detection, but that ionized calcium values would be reported proportionally to the level of dilution. Therefore, it was expected that ionized calcium values in diluted samples would be 50% of the value reported for undiluted samples.

## 2. Materials and Methods

Two groups of animals were included in this study. One group consisted of 12 eastern box turtles (*Terrapene carolina carolina)* which presented as injured wildlife to the North Carolina State University's Turtle Rescue Team (NCSU TRT) during the summer of 2016. Turtles weighing over 350 grams without visible clinical signs of major illness or injury were eligible for inclusion in the study. These animals had received treatment for injuries or illness prior to the study. However, at the time of sampling, all turtles were given a physical exam and deemed ready for release by the NCSU TRT, and therefore, free of visible clinical signs consistent with disease or injury. The sample population included seven males and five females, of adult size but unknown age, with weights ranging between 345 and 500 grams. This portion of the study was approved by the North Carolina State University Institutional Animal Care and Use Committee, protocol number 14-070-O.

The second group consisted of 12 marine toads (*Rhinella marinus*). These toads were wild-caught as part of a routine invasive species management program from a free-ranging feral population on the grounds at a zoological institution in Miami, Florida, USA. Toads weighing greater than 125 grams with no visible clinical signs of systemic disease were eligible for inclusion in the study. All toads had small iatrogenic wounds at the time of sampling as concurrent samples were being collected for a parallel wound healing study. The sample population included nine males and three females (sex was not determined until after sampling) with weights ranging between 129 and 440 grams. This portion of the study was approved by the North Carolina State University Institutional Animal Care and Use Committee, protocol number 17-199-O.

### 2.1. Sample Collection and Processing

#### 2.1.1. Box Turtles

Turtles were held in manual restraint for blood collection. A 0.5–1.0 mL blood sample was obtained from the subcarapacial sinus using a heparinized syringe. Syringes were heparinized by withdrawing heparin into the syringe and then depressing the plunger completely. Air was then withdrawn into the syringe and plunger depressed to leave scant visible heparin within the syringe. Samples containing obvious lymph contamination, characterized by transparent fluid entering the syringe first, were discarded, and new samples were collected. Undiluted heparinized blood was immediately loaded into two i-STAT CHEM8+ cartridges and analyzed on the same i-STAT machine one after the other. Measured analytes incorporated into the cartridge include sodium (Na), potassium (K), chloride (Cl), TCO_2_, ionized calcium (iCa), glucose (Glu), urea nitrogen (BUN), creatinine (Crea), and hematocrit (Hct) as well as calculated anion gap and hemoglobin (Hgb). The remaining portion of the blood sample was diluted 1 : 1 with sterile distilled water and mixed. Immediately after mixing the sample, two additional i-STAT CHEM8+ cartridges were loaded with the diluted heparinized sample and analyzed for a total of four analyses per turtle. All four analyses occurred within approximately 10 minutes of blood collection.

#### 2.1.2. Marine Toads

Toads were anesthetized for euthanasia with MS-222 (Western Chemical Inc., Ferndale, WA 98248, USA) at a dose of 5 grams/liter buffered 1 : 1 by weight with sodium bicarbonate. Upon successful anesthesia, a 0.5–1.0 ml blood sample was obtained from the sciatic vein using a heparinized syringe. Syringes were heparinized as above for turtle blood sample collection. Samples containing lymph contamination, as noted above, were discarded and new samples were collected. Undiluted heparinized blood was immediately loaded into two i-STAT CHEM8+ cartridges and analyzed on the same i-STAT machine one after the other. Measured analytes were identical to those listed above. The remaining portion of the blood sample was diluted 1 : 1 with sterile distilled water and mixed. Immediately after mixing the sample, two additional i-STAT CHEM8+ cartridges were loaded with the diluted heparinized sample and analyzed, for a total of four analyses per toad. All four analyses occurred within approximately 10 minutes of blood collection.

### 2.2. Statistical Analysis

Data were collected and entered into Microsoft Excel 2018 (Microsoft Corporation, Redmond, WA 98052 USA). Data were analyzed using R statistics (version 3.6) [21]. Data were evaluated for intraclass reproducibility across the sets of replicate measurements using the R package irr (version 0.84.1) [22]. For other analyses, replicate measures for each animal, when present, were averaged, and statistics were conducted for one data point for each animal. Data were evaluated and compared on the assumption that diluted analyte results would be half of the undiluted analyte. Fold change ratios were calculated as diluted versus undiluted measures. Collective turtle and toad data were analyzed using simple linear regression for a fit of the data and compared to what would be expected (slope of 0.5 and intercept of 0), and further examined for error and bias with both Bland–Altman plots [23] and Passing-Bablok regression analysis [24]. Passing-Bablok regression analysis was implemented using the R package mcr (version 1.2.1) [25].

## 3. Results

Several undiluted samples from both animal groups had unreported values, either due to values below the limit of detection for the analyzer or analyzer error during analysis. Ionized calcium, glucose, and total carbon dioxide were the most consistently reported values across all sample groups and the only reported values for both groups of diluted samples. The sample number, mean, standard deviation (SD), median, minimum, and maximum values for undiluted and diluted turtle samples are found in Table 1 and results from undiluted and diluted toad samples are found in Table 2. Summary statistics for diluted toad and turtle samples were limited to ionized calcium and glucose.

**Figure 1:**
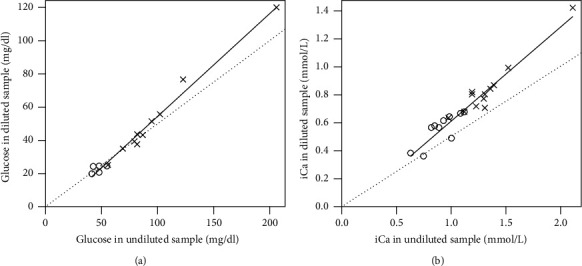
Linear regression of eastern box turtle (*Terrapene carolina carolina*) (x) and marine toad (*Rhinella marinus*) (o) samples (solid line) versus expected (dashed line) for (a) glucose and (b) ionized calcium. Linear regression slopes and nonzero intercepts for glucose were 0.62 and −7.6, respectively, and for ionized calcium were 0.68 and 0.060, respectively.

**Figure 2:**
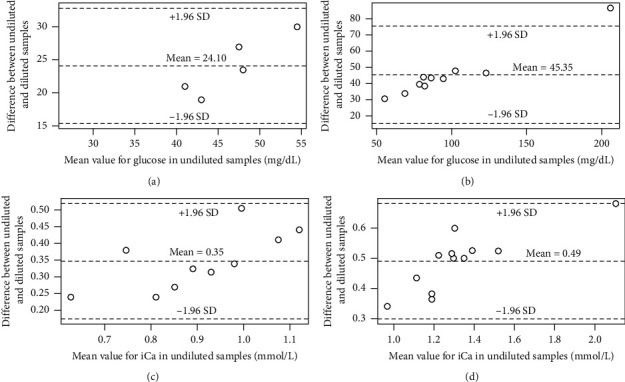
Bland–Altman plots showing the difference between undiluted and diluted samples compared to mean value of undiluted samples, for marine toad (*Rhinella marinus*) (a, c) and eastern box turtle (*Terrapene carolina carolina*) (b, d), and for glucose (a, b) and ionized calcium (c, d). Lines of agreement are shown as ±1.96 standard deviation (SD).

The reproducibility of measurements was high for all analytes measured, with the intraclass correlation coefficients ranging from 0.93 to 1.00. The ratio of diluted to undiluted values was expected to be 0.5, but all except one assay was higher than 0.5. For turtle blood, these dilution ratio values were 0.523 (95% CI: 0.485, 0.560) for Glu and 0.630 (95% CI: 0.602, 0.658) for iCa. For toad blood, these dilution ratio values were 0.487 (95% CI: 0.425, 0.550) for Glu and 0.616 (95% CI: 0.564, 0.668) for iCa.  Figure 1 shows a simple linear regression analysis of Glu and iCa, with results consistently greater than expected for iCa (i.e., diluted concentration measurements greater than 0.5x undiluted concentration measurements). Bland–Altman plots (Figure 2) display proportional error for diluted Glu and iCa concentrations compared to undiluted values. This is visually apparent because the difference between diluted and undiluted values is greater at higher concentrations of each analyte.

Passing-Bablok regression analysis was conducted due to the identified outlier (Figure 2(b)) and to further analyze for the presence of error and proportional error. A constant error was found for Glu (estimated intercept of −6.61 and 95% CI of −12.26 to −0.25), but not for iCa (estimated intercept of −0.034 and 95% CI of −0.19 to 0.14). Proportional constant error was evident for both, with slope estimations of 0.60, 95% CI [0.50, 0.67] and 0.66, 95% CI [0.50, 0.77] for Glu and iCa, respectively.

## 4. Discussion

This study evaluated the reproducibility and effects of a single dilution on reptile and amphibian blood samples using the i-STAT portable chemistry analyzer. Most of the expected analytes were consistently identifiable and measurable in undiluted samples using the portable analyzer with the exception of creatinine, which was only reported in four sample analyses. Creatinine is not considered diagnostic in reptiles due to the negligible amount produced in reptilian kidneys, consistent with results from this study [26, 27]. As expected, the utility of dilution to obtain meaningful results regarding Na+, K+, Cl-, BUN, and calculated AG appears minimal, presumably as a 1 : 1 dilution drives the analytes below the level of detection for the analyzer. TCO_2_ was reported for most of the diluted samples, but the clinical relevance of TCO_2_ values in a venous sample exposed to room air before analysis likely preclude interpretation. Furthermore, Hgb and Hct are unlikely to be representative of true values given than reptiles and amphibians possess nucleated red blood cells and the values from the iSTAT are measured and calculated based on assumptions made for human hemoglobin and red blood cells. Previous research in fish has shown that i-STAT hematocrit and hemoglobin results were inconsistent and difficult to correct for, supporting the assumption that Hct and Hgb measured in this study are also unreliable [28].

Dilution had an unexpected but reliable effect on iCa. In humans, hemodilution is reported to potentially cause an increase in measured iCa. However, this was only reported for samples diluted greater than 20% with normal saline or lactated ringers solution [29]. In this study, the expected dilution ratio was 0.50, but the mean observed ratio was 0.630 for turtles and 0.616 for toads. The average of the ranges for overestimations was 6% for turtles and 10% for toads. Considering this margin of error, individual values should be interpreted with caution, but clinical use of this dilution factor may give a general impression of the ionized calcium status of a patient. If a resulting value is below the limit of detection following dilution, extrapolation via the limit of detection for the analyzer (0.25 mmol/L for the i-STAT) multiplied by the dilution factor (1.63 for a turtle sample for example) would imply the iCa of that patient was below 0.40 mmol/L (CI: 0.37–0.43 mmol/L). Normal iCa values have not yet been established in the eastern box turtle. However, based on available normal iCa reference intervals in a related species, the ornate box turtle (*Terrapene ornate*), values less than 0.8 mmol/L would be considered abnormally low and require clinical intervention [30].

Glucose values obtained from diluted samples were closest to the hypothesized dilution ratio of 0.50, with mean observed ratios of 0.523 and 0.487 for turtles and toads, respectively. For turtles, diluted values were on average found to be within 7% of expected values, while for toads, diluted values were, on average, found to be within 13%. These levels of variation are about 1-2x greater in magnitude than the expected analytical error for i-STAT glucose measurements conducted upon calibrating standards (ranging from −7.62% to 4.82%) [31]. As related to intra-assay variability, values reported for %CV glucose (Tables 1 and 2) occur within <2x the range reported for quality controls using i-STAT (0.597% to 3.82%) [31]. For iCa and other small ions (Na+, K+, and Cl-), turtles had lower %CV than toads, yet for larger molecules such as glucose, creatinine, hematocrit, and hemoglobin, turtles had higher %CV than toads. Interestingly, CV values for undiluted samples were approximately half those of the diluted samples. Our findings indicate limited precision for dilution-based assays of blood analytes, but these limitations may not preclude clinical utility, especially if considering the monitoring of trends. As a standalone assay, handheld glucometers may be a reasonable alternative when considering sample volume constraints as they are able to use a single drop of blood for analysis. However, validation is recommended before the interpretation of results as reptilian blood glucose is often 20% higher than reported values and margin of error increases towards the lower limits of detection [32].

A limitation of this study was the sample size of both animals and samples run per animal. Increasing the population tested would have likely improved the precision and accuracy of the reported dilution ratio values. This study also focused on two relatively healthy populations of animals. Further investigation into the dilution effect on samples from animals across variable age classes, clinical health status, and reproductive status may further elucidate the clinical utility. A study investigating temporal variance in eastern box turtle blood chemistry values found that reproductive females had higher total calcium during the nesting season, and many samples were above the limit of detection for the analysis [33]. Additionally, limits of detection of the analyzer itself prohibit interpretation of values in diluted samples with the exception of iCa, Glu, and potentially TCO_2_. Another limitation of this study is the use of water as a diluent, which may place osmotic stress on erythrocytes and lead to hemolysis. However, previous research has shown that erythrocyte tolerance of osmotic stress is higher in reptiles and amphibians than mammals, and varies significantly by species [34, 35]. Amphibians, in particular, have been shown to have cells that are adaptable to differing osmolalities, resulting in variable erythrocyte osmotic fragility [34]. Presently the osmotic fragility of *T. carolina carolina* and *B. marinus* blood cells is unknown, and future investigation is needed to determine species-specific data. Additional work with the dilution of blood samples may also involve evaluating different diluents such as isotonic saline.

Validation and establishing specific reference ranges for the i-STAT analyzer by species is recommended to provide more consistent and interpretable results. A study compiling validation research for point of care analyzers across vertebrates concluded that while reported values often differed across analyzers, the values were consistently increased or decreased on machines such as the i-STAT and, therefore, can be useful as a general guide if analyzer-specific reference ranges are developed [36].

## 5. Conclusions

Based on the results of this study, sample dilution with sterile water to compensate for small sample volumes may not preclude the interpretation of glucose and ionized calcium values obtained using an i-STAT analyzer. Results should be interpreted cautiously, however, and with regard to the clinical presentation of the patient and institution-specific established references for these analytes.

## Figures and Tables

**Table 1:** Biochemistry results from heparinized undiluted and heparinized dilute blood samples from 12 eastern box turtles (*Terrapene carolina carolina*) using an i-STAT portable analyzer.

Analytes^d^	Undiluted samples								Diluted samples							
	*N* ^a^	*n* ^b^	Mean	SD	Median	Min	Max	%CV^c^	*N* ^a^	*n* ^b^	Mean	SD	Median	Min	Max	%CV^c^
Na^+^ (mmol/L)	24	12	128.83	7.4	130.5	108.5	136	0.37	—		—	—	—	—	—	—
K^+^ (mmol/L)	24	12	3.34	0.64	3.23	2.55	4.7	1.91	—		—	—	—	—	—	—
Cl^−^ (mmol/L)	24	12	97.17	7.79	99.75	75	104	0.74	—		—	—	—	—	—	—
iCa (mmol/L)	23	12	1.33	0.28	1.29	0.97	2.1	2.26	24	12	0.84	0.21	0.81	0.63	1.43	3.74
TCO_2_ (mmol/L)	24	12	23.96	6.59	24.25	8	33	13.54	—		—	—	—	—	—	—
Glu (mg/dL)	22	11	93.86	42.19	82	53.5	206	3	19	10	52.55	27.31	43.25	25	119.5	4.85
BUN (mg/dL)	22	11	48.36	30.19	43.5	10	99.5	2.12	—		—	—	—	—	—	—
Crea (mg/dL)	2	1	0.85	NA	0.85	0.85	0.85	8.32	—		—	—	—	—	—	—
Hct (%)	7	4	15.75	3.77	16	11	20	9.84	—		—	—	—	—	—	—
Hgb (g/dL)	7	4	5.34	1.3	5.42	3.7	6.8	9.8	—		—	—	—	—	—	—
AG (mmol/L)	24	12	10.58	3.84	10.5	3	16	18.77	—		—	—	—	—	—	—

^a^Some samples did not have reportable results out of the 24 samples analyzed. ^b^Number of animals assayed, for which measurement values are averaged and statistics calculated. ^c^Intra-assay coefficient of variation. ^d^Na^+^ = sodium; K^+^ = potassium; Cl^−^ = chloride; iCa = ionized calcium; TCO_2_ = total carbon dioxide; Glu = glucose; BUN = blood urea nitrogen; Crea = creatinine; Hct = hematocrit; Hgb = hemoglobin; AG = anion gap.

**Table 2:** Biochemistry results from heparinized undiluted and heparinized dilute blood samples from 12 marine toads (*Rhinella marinus*) using an i-STAT portable analyzer.

Analytes	Undiluted samples								Diluted samples							
	*N* ^a^	*n* ^b^	Mean	SD	Median	Min	Max	%CV^c^	*N* ^a^	*n* ^b^	Mean	SD	Median	Min	Max	%CV^c^
Na^+^ (mmol/L)	20	10	109.9	3.45	110.25	104	115	1.2	—	—	—	—	—	—	—	—
K^+^ (mmol/L)	20	10	3.62	0.9	3.65	2.2	4.9	4.47	—	—	—	—	—	—	—	—
Cl^−^ (mmol/L)	20	10	80.65	6.79	79	69	89.5	0.98	—	—	—	—	—	—	—	—
iCa (mmol/L)	18	10	0.9	0.15	0.91	0.62	1.12	3.31	23	12	0.55	0.10	0.57	0.37	0.68	3.45
TCO_2_ (mmol/L)	23	12	23.92	5.84	23.75	14	33	2.1	—		—	—	—	—	—	—
Glu (mg/dL)	24	12	39.5	8.66	41.25	27	54.5	0.97	9	5	22.7	2.25	24	20	24.5	5.19
BUN (mg/dL)	20	10	12.2	4.98	11.75	3	20	2.46	—	—	—	—	—	—	—	—
Crea (mg/dL)	2	1	0.2	-	0.2	0.2	0.2	—	—	—	—	—	—	—	—	—
Hct (%)	16	8	21.62	3.58	21.25	17	28.5	5.12	—	—	—	—	—	—	—	—
Hgb (g/dL)	16	8	7.36	1.22	7.22	5.8	9.7	5.13	—	—	—	—	—	—	—	—
^**AG (mmol/l)**^			20	10	^a^10	^a^6.5	^a^14.5	^a^22.96	—	—	—	—	—	—	—	—

^a^Some samples did not have reportable results out of the 24 samples analyzed. ^b^Number of animals assayed, for which measurement values are averaged and statistics calculated. ^c^Intra-assay coefficient of variation. ^d^Na^+^ = sodium; K^+^ = potassium; Cl^−^ = chloride; iCa = ionized calcium; TCO_2_ = total carbon dioxide; Glu = glucose; BUN = blood urea nitrogen; Crea = creatinine; Hct = hematocrit; Hgb = hemoglobin; AG = anion gap.

## Data Availability

OSF data are available at https://osf.io/v7cq4/?view_only=12734f2100d9476db2dfc955f2656dbe and can be made public after peer review.
